# A Study on the Photopolymerization Kinetics of Selected Dental Resins Using Fourier Infrared Spectroscopy (FTIR)

**DOI:** 10.3390/ma15175850

**Published:** 2022-08-25

**Authors:** Mirosław Kwaśny, Jakub Polkowski, Aneta Bombalska

**Affiliations:** 1 Institute of Optoelectronics, Military University of Technology, S. Kaliskiego 2 Str., 00-908 Warsaw, Poland; 2 Faculty of Mechanical Engineering, Military University of Technology, S. Kaliskiego 2 Str., 00-908 Warsaw, Poland

**Keywords:** photopolymerization, dental resin, degree of conversion, FTIR, LED

## Abstract

The aim of the presented study was a comparative analysis of the polymerization kinetics of dental resin-based composites currently used in dentistry in different environmental conditions (irradiance, activation time, layer thickness). The photopolymerization kinetics of eleven dental resins were investigated using a Woodpecker LED source. The DC was measured by FTIR in transmission mode and attenuated total reflection (ATR) from 5 s to 7 days. In the transmission mode, the spectra from parallel optical layers (about 0.2 mm thick) of samples placed between the KBr crystals were recorded. In the reflection mode, an ATR attachment with a diamond window was used. The DC calculation method was applied based on the application of a monomer absorption band at 1638 cm^−1^ (stretching vibration double bond C=C of the vinyl group) without using a reference band. The data were analyzed by performing an ANOVA test comparison between sample groups at the significance level α = 0.05. For all tested materials, the polymerization kinetics consist of three stages. The fastest stage occurs during the irradiation, and the achieved DC value is 70–75% of the maximum value 5 s after the irradiation. Another 15–20% DC increase at a moderate speed takes about 15–20 min. There is also a very slow further increase in DC of 5–10% within 5 days after irradiation. For 8 out of the 11 tested fillings, the optimal photopolymerization conditions are as follows: a power density of 400 or 1000 mW/cm^2^; an exposure time of 10 s; and a thickness of the irradiated resin layer of up to 2 mm. The influence of various conditions and factors on the reaction kinetics is dominant only in the early, rapid phase of the conversion. After longer times, the DC values gradually level out under different light conditions. The DC of the dental resins are dependent on the irradiance, light source, filler type, time after irradiance, and monomer thickness.

## 1. Introduction

The composite resin used in dentistry can be divided into the following groups: macrofilled, microfilled, nanofilled, hybrid, and short fiber-reinforced composite [1,2]. These materials contain filler particles with diameters between 10–100 μm; they are hard and difficult to polish [3]. Resin has particles between 0.04–0.2 μm and a high polish ability [4]. The combination of microfilled and macrofilled is called “hybrid “composite resin. It contains a colloidal silica particle size of 0.01–0.05 μm and has a smooth polishing surface, a good wear resistance, and good mechanical properties [5]. Nanofilled composite resin includes zirconium/silica and nanosilic particles with a diameter of 25 nm and a silanized agglomerate size of 75 nm. The filler content is up to 80%, leading to reduced polymerization shrinkage and increased mechanical properties [6].

In dentistry, the monomer in the form of methyl methacrylate derivatives is used in almost all types of resins, and the polymerization process is vinyl-free-radical. Composite resin is classified into chemically activated and light activated [7,8,9].

The degree of monomer conversion (DC) in dental fillings based on composite resins is a very important parameter. Due to the introduction of new materials into the market, the problem of DC determination is still the subject of a large amount of current research.

The DC depends on many factors such as the matrix composition, the intensity of irradiation, the light transmission properties of the matrix, the filler particle type and size, the concentration of the polymerization catalyst, and the type of photo-initiator system [10].

The DC value directly influences the mechanical properties, resistance, polymerization shrinkage, water sorption, and solubility of the irradiated resins. Low DC values result in increased cytotoxicity and decreased material hardness. DC values that are too low may result in the release of unreacted cytotoxic monomers [11]. The complete conversion of bifunctional methacrylates is never attainable because diffusional restrictions in later stages of the polymerization reaction prevent a certain number of monomer molecules from reaching reaction sites [1].

Depending on the photopolymerization parameters of the matrix type, the maximum DC values reported in the literature are in the range of 40–70% [12,13,14]. The main reason for the discrepancies in the DC literature of the same materials is the failure to consider the polymerization time of the sample after irradiation and the use of different measurement techniques. 

So far, the following methods have been used to determine the DC: Fourier infrared absorption spectroscopy (FTIR) [15,16,17,18,19,20,21,22], Raman spectroscopy [23,24], electron paramagnetic resonance (EPR) [25], nuclear magnetic resonance (NMR) [26], and differential scanning calorimetry (DTA) [27,28].

FTIR spectroscopy is one of the most used methods, but the literature results relate to measurements carried out under various conditions, and, so far, no standardized method has been proposed. Polymer samples can be prepared in various forms. Usually, they are powdered and then mixed with a dry potassium bromide (KBr) powder [29,30,31]. In recent years, the attenuated total reflection (ATR) sampling technique was effectively used for DC determination [13,14,32,33,34,35,36]. This technique is characterized by its simplicity; samples are examined directly in the solid or liquid state without further preparation. In the case of real-time experiments, a monomer drop is sandwiched between NaCl or KBr plates and then polymerized directly in a measuring chamber of the apparatus [37,38,39,40]. In publications, absorption spectra are presented in exceptional cases, which is rare in typical spectroscopic analyses. Some publications have critically assessed the work to date on the determination of DCs, e.g., [41]. 

The current methods used in determining DC are based on comparing the absorption bands that are typical of the carbon double bond (C=C, 1638 cm^−1^) of the monomer and one of the reference bonds. The most used reference absorption band has the wavenumber of 1608 cm^−1^, although others such as 1730 or 4623 cm^−1^ are also used [18]. However, this method is correct if the reference band exhibits this intensity for the entire DC range. This, however, as we have shown, is not accurate for any methyl methacrylate derivative. In the transmission mode, the principles of the method are presented in [10]. 

This DC calculation method has an additional systematic error of at least 5% when the 1608 cm^−1^ reference band is used. Moreover, as we will show, there are resins for which this reference band is barely visible. We present the most reliable method in which reference bands are not necessary. 

In the present study, the kinetics of resin polymerization were investigated in two measurement modes—transmission and reflection (ATR). The transmission method consists of examining the optical polymerization of the resin layer placed between the KBr crystals. The entire cross-section of the light beam from the optical fiber falls perpendicularly on the crystal surface, and the polymerization process of the same sample is measured at any time, from 0 s to even weeks after exposure. The degree of polymerization is determined by measuring the intensity decrease of the methacrylate absorption band at 1638 cm^−1^, which relates to the C=C stretching vibration of the vinyl group.

This method is well suited for limited aging tests; however, it has a sample thickness of up to 0.2 mm due to the strong absorption of resins. The obtained DC values are averaged for the entire layer thickness. In this method, the DC values are not influenced by the contraction of the resin layer thickness. The ATR method allows for DC measurements of any sample thickness, but the absorption signals relate to a layer thickness of several micrometers due to limited light penetration.

The main purpose of the work was to test and compare the DC values of currently used dental resins under various experimental conditions (irradiance, exposure time, layer thickness) using a classic LED source. The kinetics of polymerization were investigated with the ATR technique, which was previously used in the works of Ilie et al. [13] and Frauscher and Ilie [14]. It was limited to a few fillings, and the polymerization was tested for a very short time—up to 5 min after irradiation. Incomplete resin conversion is an intriguing phenomenon, and its explanation is important to dental practice. The goal of this study is to enable people to understand the conversion process. 

## 2. Materials and Methods

The materials used in the study are presented in Table 1.

Dental fillings have a very diverse composition. An example of the composition of the UD2 material is described as follows: (a) monomers (25%): Diurethandimethacrylate, Iso-propyliden-bis (2(3)-hydroxy-3(2)-4(phenoxy)propyl-bis(methacrylate) (Bis-GMA), 4-Butandialdimethacrylate; (b) filler (75%): Glass filler, medium particle size of 0.7 µm, silicon dioxide, medium size—0.04 µm.

### 2.1. Methods

The blue light LED F lamp (Woodpecker, Guilin, China) designed for dental use was used to expose the samples. The source has a choice of the irradiation power density (400, 1000, and 1600 mW/cm^2^) and exposure time (10, 20, and 40 s). It is also equipped with a radiation power density meter, which is built into the charging station. The lamp has a choice of exposure time of 10 and 20 s and is also equipped with a power density meter built into the charging station.

The IS50 ATR Module FTIR spectrophotometer (Thermo Scientific, Waltham, MA, USA) was used to measure the FTIR spectra. The spectra were collected in absorbance mode in a range of 1200–1800 cm^−1^, with a 4 cm^−1^ resolution and 64 scans.

### 2.2. Preparation of the Sample

Two modes were used for the DC measurements of the resins—reflection and transmission. In the reflection mode, an ATR reflection adapter was used.

A plate of a certain thickness, in which there was a 9 mm diameter hole for the sample, was placed on the diamond crystal of the adapter. After placing the sample in the hole of the plate, it was pressed with a microscope slide with a thickness of 0.5 mm, and the optical fiber was placed directly against the slide so that its surface was exactly in line with the hole of the sample. Absorption is measured at the interface between the crystal surface and the sample, with radiation coming from the opposite side. Therefore, it is the same situation as that in the real conditions of dental treatment. The DC value is the most important at the contact between the resin and the tooth. In the ATR method, the sample must adhere to the crystal surface throughout the test. Its removal and re-application prevent proper contact and cause a significant reduction in the measured absorbance value.

**Figure 1:**
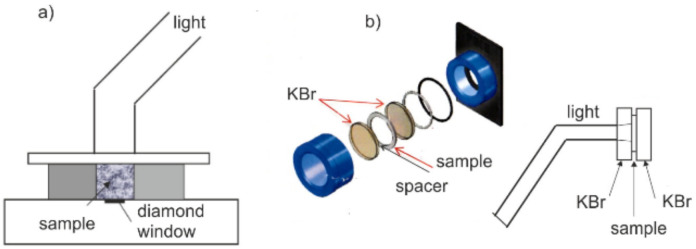
(**a**) Measurements with the ATR adapter, (**b**) Measurements in transmission mode.

In the transmission mode, the samples were placed centrally on the KBr crystal (diameter 25 mm, thickness 3 mm). A 0.2 mm spacer was placed over the periphery of the crystal, and a second KBr crystal was applied. They were slowly compressed to the configuration of the washer thickness and placed in the holder. The schematic arrangement of the sample is presented below in Figure 1.

Before irradiating this surface of the sample with a light guide (8 mm in diameter), the FTIR spectrum was measured at different times. The diameter of the spot of light reaching the monomer after 3 mm of the crystal had passed through was 9 mm, and the diameter of the diaphragm used in the spectrophotometer was the same. This means that the entire cross-section of the light beam from the optical fiber illuminated the sample and that the average FTIR spectrum was determined from the entire surface of the illuminated sample. Moreover, the lack of a homogeneous distribution of light intensity from the optical fiber was eliminated.

### 2.3. Calculation of the Degree of Polymerization Conversion

**Figure 2:**
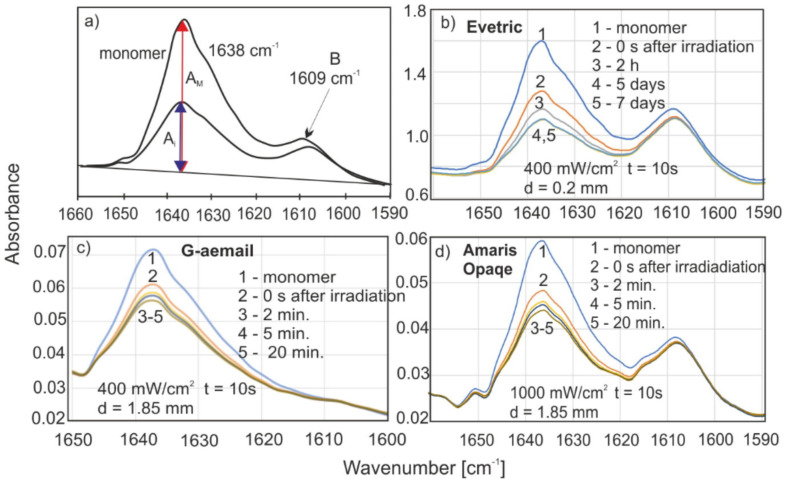
(**a**) The method of determining the value of the absorbance of the bands by taking into account the baseline, (**b**) an example of the change of the absorption spectrum in the transmission mode, (**c**,**d**) examples of the changes of the absorption spectra in the reflectance mode (ATR).

The degree of polymerization was calculated based on the monomer loss, which has a characteristic absorption band located at the wavenumber of 1638 cm^−1^ (which relates to the C=C stretching vibration of the vinyl group). The monomer concentration drops during the polymerization. The method of determining the absorbance value is shown in Figure 2a.

The degree of polymerization in the time *DC* (*t*) was calculated from Formula (1):

(1) $$DC\left(t\right)=\left(1-\frac{A_{t}}{A_{m}}\right)\cdot 100\%$$

where: *A_t_*—sample absorbance after time *t*; *A_m_*—monomer absorbance.

### 2.4. Statistical Analysis

The statistical analysis of the results was performed using the ANOVA program. The DC values are from separate experiments.

The confidence interval (*L*) for the probability *p* = 99% is:

(2) $$L=\bar{x}\pm t\cdot s\left(\bar{x}\right)$$

where: *t*—Student′s *t*-distribution coefficient for *p* = 99% and *n* = 5 (*t* = 4.02) *L* = 44.2 ± 3.1%.

For the DC of about 25%, the obtained *L* value is 25 ± 4.9%.

For all experiments, the relative measurement error was assumed to be ±5%. The presented method is very precise. The statistical deviations are the result of the polymerization process.

## 3. Results

### 3.1. Irradiation Parameters’ Impact on DC

**Figure 3:**
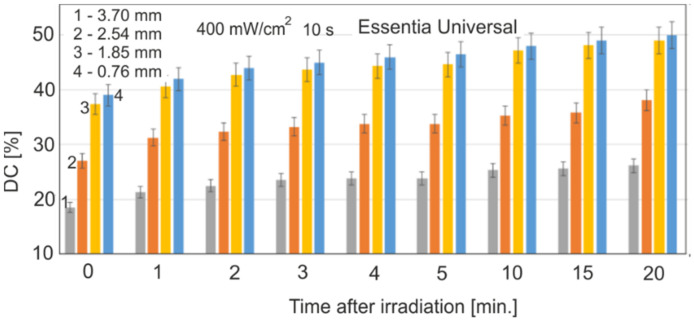
Influence of the sample thickness on the DC value.

Figure 3 shows the effect of a single layer thickness on the DC value of the Essentia Universal for an incident radiation power density of 400 mW/cm^2^ and an exposure time of 10 s, with different sample thicknesses.

The typical range of the layer for dental applications was selected as 0.76 up to 3.70 mm. There were statistically significant differences between the DC values for thicknesses of 1.85, 2.54, and 3.70 mm (*p* < 0.01). The DC values for samples with layer thicknesses of 0.76 and 1.85 mm are very similar, and there are no statistically significant differences between them (*p* > 0.3). 

**Figure 4:**
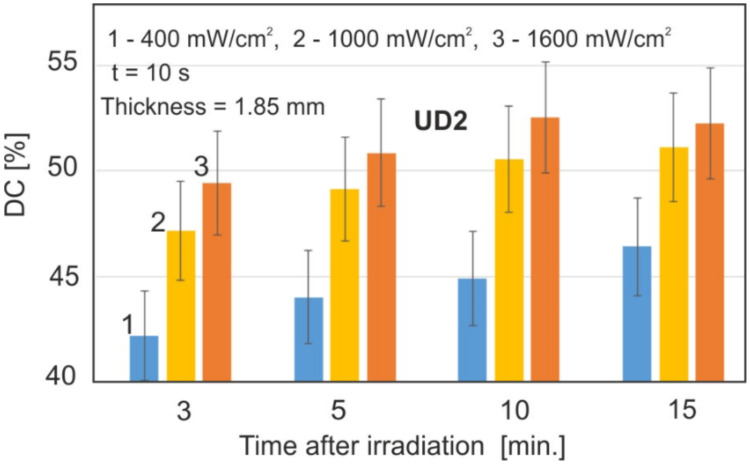
Effect of power density on DC values.

The effect of the radiation power density is shown in Figure 4. The values of 400, 1000, and 1600 mW/cm^2^ were used. The sample thickness and exposure time remained the same for all the samples.

There was a statistically significant difference between the irradiation powers (*p* < 0.05). Based on the obtained results, it was found that the most optimal values will be 400 and 1000 mW/cm^2^; the value of 400 mW/cm^2^ was chosen due to the slower rate of the polymerization process and a clear difference in the results from 1000 mW/cm^2^.

**Figure 5:**
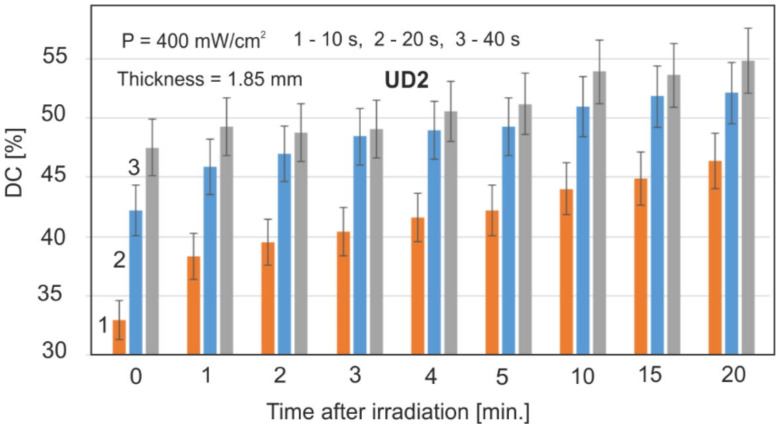
Effect of the exposure time on the DC values.

From the point of view of dental applications, the important parameter is the exposure time. Figure 5 shows the value of DC after irradiation at 10, 20, and 40 s.

### 3.2. DC Results for Individual Materials

**Figure 6:**
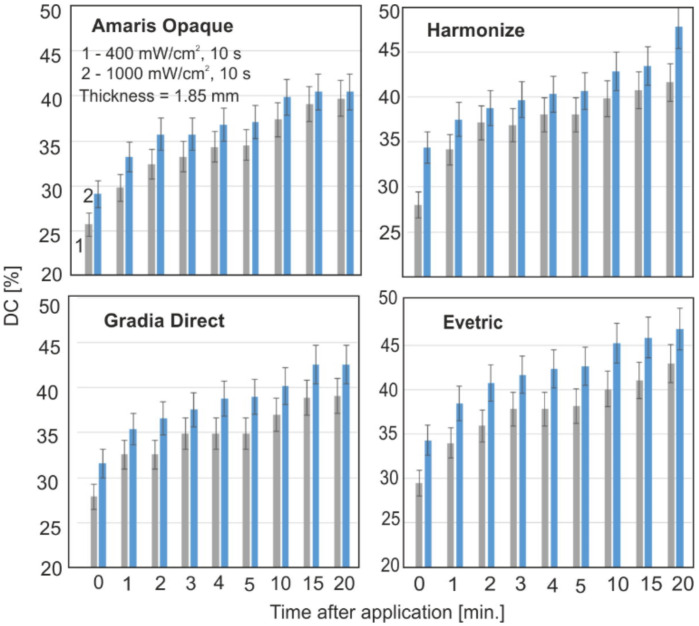
The polymerization kinetics of the materials with the average DC value.

Figure 6 show the collective characteristics of the DC change during the polymerization of the individual materials. The tests were carried out with a layer thickness of 1.85 mm and power density of 400 and 1000 mW/cm^2^. The exposure time (10 s) was selected because it is mostly used in dental practice. However, for this time, slower polymerization is observed, and it is better to compare different materials. With time, after radiation, the DC slows down. The lower irradiation power results in lower DC values; the biggest changes can be seen up to four minutes. For Filtek and G-aemail, the largest differences in DC values for different exposure powers were observed.

**Figure 7:**
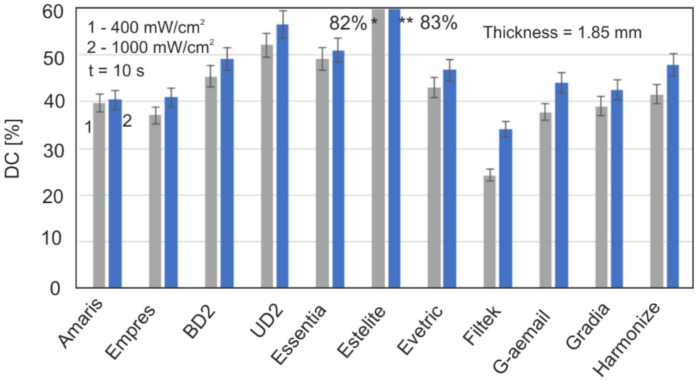
Comparison of the maximum DC values for the tested materials. * DC for Estelite (400 mW/cm^2^) ** DC for Estelite (1000 mW/cm^2^).

Figure 7 shows a comparison of the maximum DC values of all the materials 20 min after being irradiated.

After 20 min, the changes in DC are already very slow, with the order of a single percentage point per hour. They can be observed after many hours and then days. After 5 days, any changes in the DC values practically cease. The difference in DC values between polymerization times between 20 min and 5 days is up to several percentage points. This indicates how slowly the last stages of polymerization are proceeding. It can be assumed that the time of 20 min is long enough to compare the final DC values for all of the test materials.

### 3.3. Polymerization Kinetics in the Transmission Method

The presented results of the polymerization rate indicate that its fastest stage occurs during the irradiation of the sample itself. After 1 min at the end of the illumination, the DC value obtained is about 80%. Over the next 20 min, an increasingly slower process is observed, and the total DC obtained is approximately 20% of the maximum value. It can be considered that the polymerization has practically ceased 15 min after exposure, although a slight increase in DC is still observed.

The question is, at what time will such low DC gains not be observed? In the reflection technique, the sample must remain in the instrument all the time; hence, the transmission variant of the FTIR method was used for long-term aging tests.

The power density of the radiation reaching the KBr crystal on the sample was 210 mW/cm^2^. This corresponds approximately to the power density of the radiation that falls on the inner surface of the sample (d = 1.85 mm) in ATR tests with a power density of 400 mW/cm^2^ selected.

However, the actual conditions used in the two types of FTIR techniques differ. It is important to observe that, for a few days after irradiation, residual polymerization is still in progress, and the result is a further increase in DC by approximately 10%. After 5 days, no further increase in DC was observed.

## 4. Discussion

In practically all of the publications concerning the DC determination method to date, a point measurement with the use of a reference band is used. Figure 2 clearly shows that the reference absorption band at the wavenumber of 1608 cm^−1^ is not constant during the polymerization in its initial stages.

The reference point in the DC calculation is the monomer absorbance peak, so a systematic error is made over the entire DC range. The measurement and reference bands are not separated. The condition for such separation is the location of point B (Figure 2a) on the baseline. This is a classic case in spectroscopic research. The value of the actual absorbance of the reference band can be determined by the deconvolution method. There are also cases of resins where the absorption band at 1608 cm^−1^ is barely visible (Figure 2c), and, in these cases, the method based on relative measurements would not be possible in point measurements.

The method used to determine the DC value based on the polymerization kinetics during the same sample is very accurate and is independent of any reference bands. The reference band is strongly influenced by the dominant measuring band; on the other hand, the influence of the reference band on the measuring band is negligible.

Figure 2b shows the change in the absorbance spectrum of an exemplary resin (Evetric) for the transmission mode. The absorbance values for a layer thickness of 0.2 mm are from 0.8 to 1.6 in the wave number range of 1590–1650 cm^−1^.

Analogous graphs of the polymerization kinetics for the reflectance mode of selected resins are shown in Figure 2c,d. The penetration of IR radiation is several micrometers; hence, the absorbance in this optical path is 0.02–0.07. So, compared to the transmission technology, it is several dozen times smaller, and the signal/noise ratio is also lower. The transmission technique is unfortunately limited by the thickness of the measured resin layer to about 0.5 mm.

The power density activating the photopolymerization of the radiation is of fundamental importance for the DC values. It is especially visible in the initial stages of monomer conversion; with time, these differences become smaller and smaller. However, the increase in power density from 1000 to 1600 mW/cm^2^ does not cause a significant increase in DC (Figure 4).

It can be assumed that the power density of 1000 mW/cm^2^ is the limit above which the maximum DC values are reached in a sufficiently long time, and further increasing the power does not increase the DC, In general, the power density affects the speed of the polymerization process, especially in the initial stages; but the difference in the final DC values between the irradiations of 400 and 1600 mW/cm^2^ is about 5%. For longer times after the initiation of polymerization, e.g., hours or days, these differences become even smaller.

The values of the power density of the radiation reaching the end of the sample depend on the thickness of the resin layer. With the increase in its thickness, the radiation intensity decreases exponentially due to the absorption phenomenon and, in this case, the dominant scattering effect. The research on the effect of thickness was carried out for the range typical for practical dentistry (0.75–3.5 mm) (Figure 3).

The effect of the significant thickness on DC is observed at greater thicknesses from 2.5 mm upwards. For layer thicknesses below 2 mm, the intensity of the incoming radiation is high enough, and this effect is negligible for most materials. There is therefore a limit value for the power density of the incoming radiation, above which the DC value no longer increases.

An important parameter is also the exposure time related to the amount of light dose. As could be expected, with the increase in the exposure time, the DC values increase (Figure 5). It is also especially visible in the initial stages of polymerization. Later, these DC values become more and more similar.

The difference in the maximum DC values between the extreme times of 10 and 40 s after 20 min of polymerization is only 7% and will continue to decrease over time. With the exposure time of 10 s, the polymerization speed is comparatively lower only in the initial stage, which is advantageous due to the formation of lower mechanical stresses on the material.

Modern LED sources have the option of a gradual increase in irradiance just to slow down these initial rapid processes.

The kinetics of all the tested resins from 5 s to 20 min showed that the highest DC values (about 50% and more) were achieved by materials such as Estelite, Essenta, UD2, and BD2. Estelite had a unique DC value of over 80%, while the rest of this group does not exceed 60%. Filtek, Empress, and G-aemail had the lowest values (DC below 45%). For the other materials, the DC value does not exceed 50%.

Filtek, Empress, and G-aemail had lower DC values because their layer thickness was too high. Increasing the radiation density to 1600 mW/cm^2^ or reducing the layer thickness to 1 mm causes an increase in DC by about 10%.

For all the materials, with different dose irradiation polymerization conditions and film thicknesses, similar polymerization rate characteristics are observed up to 15–20 min after irradiation. The highest speed occurs during the irradiation itself, and after 5 s from its completion, the DC values become 75–80% of the maximum achieved after 20 min.

The remaining time, 15–20 min, has only 20–25% of the maximum value, which indicates a much slower stage. The time, 15–20 min after irradiation, could practically be considered as final, after which the DC values do not change noticeably in the following hours.

**Figure 8:**
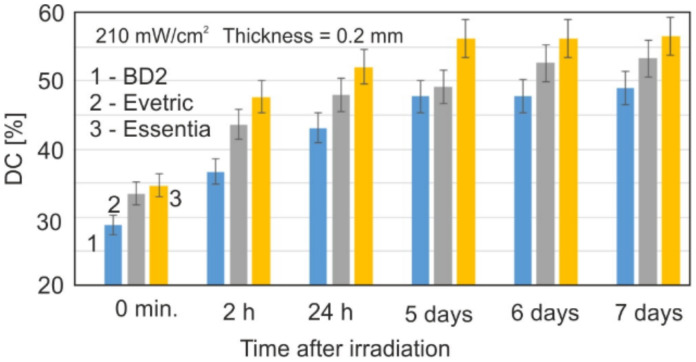
Changes in the DC values with the aging time for irradiated dental resin samples.

However, very small changes in the DC were observed; hence, this extended the testing of samples over time, even up to 7 days after irradiation (Figure 8). Within 5 days, a further increase in DC by about 10% was observed in relation to the 20 min time originally considered as final. This is a very important observation, indicating a very slow termination process of polymerization.

## 5. Conclusions

In general, it can be stated that, in the vast majority of the final materials, the achieved DC value is in the range of 45–60%. In the literature, such small DC values are explained by the overly low mobility of the polymer chain ends. It seems that the obtained DC values of all the tested materials are simply related to too little of an activator.

From the application point of view, it can be concluded that the parameters—a power density of 400 mW/cm^2^, an exposure time of 10 s, and a layer thickness of up to 2 mm—are optimal in clinical conditions. Increasing the strength or dose will not significantly affect the final DC values. For those materials with the lowest DC values, however, the maximum layer thickness should be reduced to about 1 mm, and a power density of 1000 mW/cm^2^ should be used.

For optimal thicknesses and power density, they level out after a few days. For materials such as Filtek, this DC equalization on both surfaces at a thickness of 1.85 mm was not observed. This is a clear indication that the thickness was too thick.

## Figures and Tables

**Table 1:** The dental resins used in this research.

Sample Name	Manufacturer
Enamel Plus HRi (UD2)	Micerium S.p.A., Avegno, Italy
Enamel Plus HRi BioFunction (BD2)	Micerium S.p.A., Avegno, Italy
Estelite Asteria	Tokuyama Dental Corporation, Tokyo, Japan
Filtek^TM^ Ultimate	3M Oral Care, Irwindale, CA, USA
Essentia	GC United Kingdom Ltd., Newport Pagnell, UK
Gradia Direct Anterior	GC United Kingdom Ltd., Newport Pagnell, UK
IPS Empress Direct	Ivoclar Vivadent, Buffalo, NY, USA
G-aenial	GC United Kingdom Ltd., Newport Pagnell, UK
Evetric	Ivoclar Vivadent, Buffalo, NY, USA
Harmonize^TM^	Kerr, Orange, CA, USA
Amaris	VOCO, Cuxhaven, Germany

## Data Availability

Not applicable.
